# Molecular Diagnosis of Encephalitis/Meningoencephalitis Caused by Free-Living Amoebae from a Tertiary Center in India

**DOI:** 10.3390/pathogens11121509

**Published:** 2022-12-09

**Authors:** Sumeeta Khurana, Chayan Sharma, Bishan Dass Radotra, Abhishek Mewara, Parveen Tanwar, Priya Datta, Rakesh Sehgal

**Affiliations:** 1 Department of Medical Parasitology, Postgraduate Institute of Medical Education & Research, Chandigarh 160012, India; 2 Department of Histopathology, Postgraduate Institute of Medical Education & Research, Chandigarh 160012, India

**Keywords:** *Acanthamoeba*, *Balamuthia*, central nervous system, encephalitis, free-living amoeba, meningitis, *Naegleria*

## Abstract

Background: Pathogenic free-living amoeba (FLA) such as *Acanthamoeba* spp., *Naegleria fowleri*, and *Balamuthia mandrillaris* are causative agents of fatal amoebic encephalitis/meningoencephalitis. The diagnosis of such infections is challenging due to a lack of clinical suspicion and expertise in microscopic identification. We evaluated the performance of molecular assays for the timely and accurate detection of FLA-causing central nervous system (CNS) afflictions. Methods: This study included samples from 156 patients with suspected encephalitis/meningoencephalitis, including 149 cerebrospinal fluid (CSF) samples, 5 brain tissue biopsies, and 2 brain abscess samples. All the samples were subjected to PCR-based detection of *Acanthamoeba* spp., *N. fowleri*, and *B. mandrillaris*. The diagnostic characteristics and the inter-rater reliability scores were evaluated for parasite-specific polymerase chain reaction (PCR) using culture on non-nutrient agar (NNA)/microscopy or histopathological examination as a confirmatory test for *Acanthamoeba* spp. and *N. fowleri* and histopathology for *B. mandrillaris*. Results: We detected 11 samples positive for FLA, including 6 *Acanthamoeba* spp., 3 *B. mandrillaris*, and 2 *N. fowleri*. Furthermore, all 11 samples were positive according to the confirmatory tests, i.e., culture on NNA/microscopy/histopathology in the case of *Acanthamoeba* spp. and *N. fowleri* and histopathology of tissue biopsies for *B. mandrillaris*. The inter-rater reliability between the PCRs and the confirmatory tests for the detection of *Acanthamoeba* spp., *N. fowleri*, and *B. mandrillaris* was 100%. Conclusions: The PCR-based detection of FLA in patients suspected of encephalitis/meningoencephalitis was found to be fast, efficient, and reliable in our study. We suggest the use of these PCRs in laboratories to obtain additional data on their efficiency in diagnosing FLA infections of the CNS. The present study was conducted with a small sample size of 156 patient samples, and we found only six *Acanthamoeba* spp., three *B. mandrillaris*, and two *N. fowleri*. The present study should be conducted on a larger sample size for better evaluation of the primer pairs.

## 1. Introduction

Central nervous system (CNS) parasitic infections are associated with significant morbidity and mortality. Free-living amoebae (FLA) are increasingly reported to cause infections of the CNS, although their exact burden remains unknown. The etiology in many cases of encephalitis remains obscure [1] due to invasive sampling, as well as difficulty in diagnosis. It is likely that a large share of such infections caused by FLA remain undetected and thus underestimated. The FLA genera implicated in causing CNS infections include various species of *Acanthamoeba* and *Balamuthia mandrillaris*, causing granulomatous amoebic encephalitis (GAE) mainly in the immunocompromised population, and *Naegleria fowleri*, causing primary amoebic meningoencephalitis (PAM) [2,3]. The prognosis of these infections is very poor, and in the absence of timely diagnosis and treatment, they are associated with >80% mortality; most cases are diagnosed postmortem. This highlights the lack of awareness and the need for early diagnosis and appropriate treatment. The treatment regimes for CNS infections due to FLA are not very well established. Drugs are usually recommended on the basis of the available literature with respect to survivors. The Centers for Disease Control and Prevention (CDC) suggest various combinations of conventional and liposomal amphotericin B, azithromycin, clarithromycin, fluconazole, ketoconazole, cotrimoxazole, pentamidine, rifampin, sulfadiazine, flucytosine, miltefosine, thioridazine, and dexamethasone for the treatment of PAM and GAE [3,4,5,6,7,8,9,10,11,12,13,14].

The diagnosis of CNS infections due to FLA is difficult, mainly due to the absence of a specific clinical manifestations/features and the lack of suspicion and expertise in identifying them microscopically [2,15]. Direct microscopic detection has low sensitivity [16,17]; moreover, it may be difficult to distinguish between various FLA based only on morphology, and culture may take longer to grow. Moreover, *B. mandrillaris* does not grow on non-nutrient agar (NNA) medium. Thus, appropriate molecular assays can allow for timely and accurate pathogen detection. In the present study, we evaluated the utility of different primers for the detection of *Acanthamoeba* spp., *N. fowleri*, and *B. mandrillaris* by polymerase chain reaction (PCR) as compared to the conventional confirmatory methods of culture, microscopy, and histopathology for patients suspected of encephalitis/meningoencephalitis.

## 2. Materials and Methods

A total of 156 patient samples (149 cerebrospinal fluid (CSF) samples, five brain tissue biopsies, and two brain abscess samples) with suspected encephalitis/meningoencephalitis received at the Department of Medical Parasitology, Postgraduate Institute of Medical Education and Research, Chandigarh, India, submitted for the detection of FLA from the year 2014 to 2022 were included in this study. This study was approved by the Institutional Ethics Committee (NK/5283/PhD/213).

The CSF, brain abscess, and brain tissue biopsies were either inoculated onto the NNA for monitoring of the growth of *Acanthamoeba* spp. and *N. fowleri* or were confirmed microscopically for the presence of FLA or DNA detection by PCR. These patient samples had been excluded for most common bacterial, fungal, and viral causes of encephalitis/meningoencephalitis or had been microscopically suspected of FLA infections. For the NNA culture, the patient samples were inoculated on NNA plates with a lawn of *Escherichia coli* and incubated at 37 °C for seven days to monitor the growth of trophozoites and cysts (NNA culture was used for *Acanthamoeba* spp. and *Naegleria fowleri*). Due to the inability of *B. mandrillaris* to grow in NNA culture, histopathology was considered the confirmatory test. In the case of *Acanthamoeba* spp. and *N. fowleri* infection, growth on the NNA medium/microscopic or histopathological demonstration of parasite was considered the confirmatory test.

### 2.1. DNA Extraction

DNA was extracted from the CSF, brain biopsy, and brain abscess samples using a DNeasy blood and tissue kit (Qiagen India Pvt. Ltd., New Delhi, India) as per the manufacturer’s instructions. The CSF samples were centrifuged at 6000 rpm for 10 min, and the pellet was washed with phosphate-buffered saline. The supernatant was discarded, and 200 µL of the sample was used for DNA extraction. Additionally, the DNA of *Toxoplasma gondii* and *Entamoeba histolytica* was used as other disease controls, and *Acanthamoeba* and *Naegleria* culture isolates were used as positive controls.

### 2.2. Polymerase Chain Reaction

All the samples were subjected to amplification of the housekeeping glyceraldehyde 3-phosphate dehydrogenase (GAPDH) gene using specific primers that amplify a product of 380 bp [18]. The samples were subjected to pan-FLA PCR that targets the *18S rRNA* gene sequence and differentiates the FLA, including *Acanthamoeba* spp., *N. fowleri*, *Vannella* spp., *Vahlkampfia ovis*, and *Hartmannella vermiformis* based on the size of their PCR product but fails to detect *B. mandrillaris* [19]. The samples found to be positive for *Acanthamoeba* spp. and *N. fowleri* by pan-FLA PCR were then confirmed by parasite-specific PCR targeting of *Acanthamoeba* spp. and *N. fowleri*. For *Acanthamoeba* spp., the JDP primers targeting the *18S rRNA* gene were used [20]. *N. fowleri* were identified using the genus- and species-specific primers targeting the ITS1 and ITS2 regions [21]. In addition to pan-FLA PCR, the samples were subjected to PCR targeting the mitochondrial *16S rRNA* gene of *B. mandrillaris* [22] and nested PCR to target the *18S rDNA* gene of *B. mandrillaris* [23]. The amplified DNA products were separated on a 1.5% agarose gel and viewed in a gel documentation system (ProteinSimple AlphaImager HP System Automated Gel Imaging, Santa Clara, CA, USA). The details of the primers used in the present study are provided in Table 1.

### 2.3. Statistical Analysis

The diagnostic sensitivity, specificity, positive predictive value, negative predictive value, and 95% confidence intervals (CIs) were calculated for samples found to be positive by parasite-specific PCRs using culture on NNA/microscopy or histopathological examination as the confirmatory test for *Acanthamoeba* spp. and *N. fowleri* and histopathology for *B. mandrillaris*. Statistical analysis was carried out using SPSS v. 16.0 (SPSS South Asia Pvt. Ltd., Bangalore, India). The inter-rater reliability was assessed for all laboratory techniques by Cohen’s kappa coefficient test.

## 3. Results

**Figure 1:**
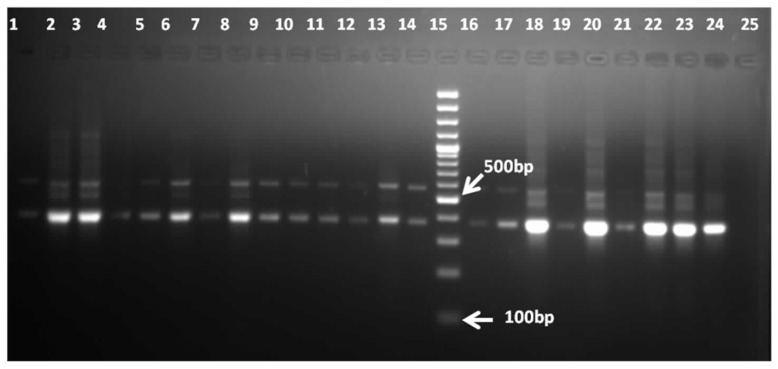
Representative image of 1.5% agarose gel electrophoresis showing the results of the amplification of the GAPDH housekeeping gene. Lanes 1–14 and lanes 16–23, samples amplifying the 380 bp GAPDH gene segment; lane 15, 100 bp molecular marker; lane 24, positive control; lane 25, negative control.

This study included a total of 156 patient samples (149 CSF samples, 5 brain tissue biopsies, and 2 brain abscess samples). All samples were positive for the housekeeping *GAPDH* gene (Figure 1).

**Figure 2:**
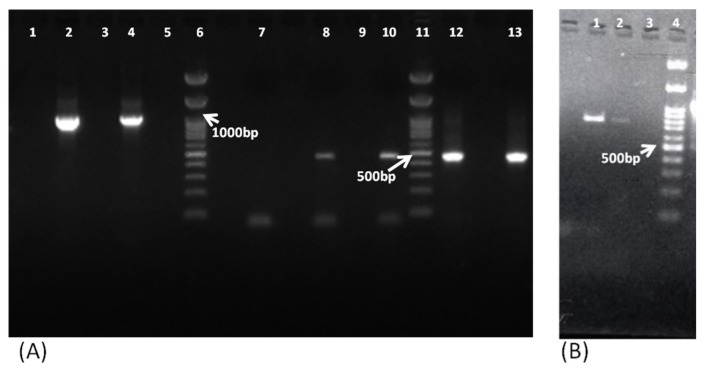
Representative images of 1.5% agarose gel electrophoresis for pan-FLA PCR and JDP PCR amplifying *Acanthamoeba* spp. and Pan-FLA PCR amplifying *N. fowleri.* (**A**) Lane 1, negative control; lane 2, positive control amplifying *Acanthamoeba*-specific 1080 bp band; lanes 3 and 5, patient sample detected as negative; lane 4, patient sample detected as positive by pan-FLA PCR; lane 6, 100 bp molecular marker; lane 7, negative control for JDP PCR; lanes 8 and 10, samples detected positive by *Acanthamoeba* spp.-specific JDP PCR; lane 9, negative patient sample; lane 11, 100 bp molecular marker; lanes 12 and 13, positive control for *Acanthamoeba* spp.-specific JDP PCR. (**B**) Lane 1, positive control for *N. fowleri* at 900 bp based on pan-FLA PCR; lane 2, patient sample detected as positive for *N. fowleri* by pan-FLA PCR; lane 3, negative control for pan-FLA PCR; lane 4, 100 bp molecular marker.

**Figure 3:**
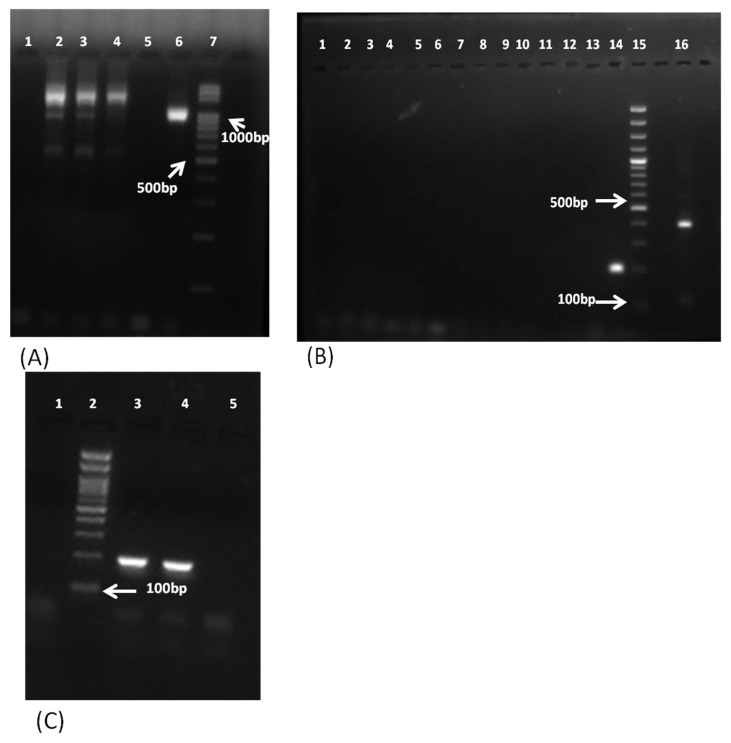
Representative images of 1.5% agarose gel electrophoresis for the *B. mandrillaris* PCR targeting the mitochondrial *16S rDNA* gene and the *18S rDNA* gene. (**A**) Lane 1, negative control; lanes 2–4, multiple bands of varying size in *T. gondii*-, *E. histolytica*-, and *N. fowleri*-positive samples; lane 5, CSF samples detected as negative; lane 6, positive control amplifying 1075 bp band; lane 7, 100 bp molecular marker. (**B**) Lanes 1–12, samples detected as negative for *B. mandrillaris* in the second PCR cycle targeting the *18S rDNA* gene; lane 13, negative control; lane 14, positive control amplifying a band of 201 bp (second PCR cycle); lane 15, 100 bp molecular marker; lane 16, positive control amplifying a band of 403 bp (first PCR cycle). (**C**) Lane 1, negative control; lane 2, 100 bp molecular marker; lanes 3 and 4, patient detected positive by *B. mandrillaris 18S rDNA* gene PCR and amplifying 201 bp band (second PCR).

Of the 156 samples, PCR detected 11 samples positive for FLA, including 6 *Acanthamoeba* spp., 3 *B. mandrillaris,* and 2 *N. fowleri*. Among these, eight samples were found to be positive by pan-FLA PCR, including *Acanthamoeba* spp. and *N. fowleri* (Figure 2), and three samples were found to be positive by mitochondrial *16S rRNA* gene PCR (Figure 3A) and *18S rDNA* gene PCR (Figure 3B) of *B. mandrillaris*.

**Figure 4:**
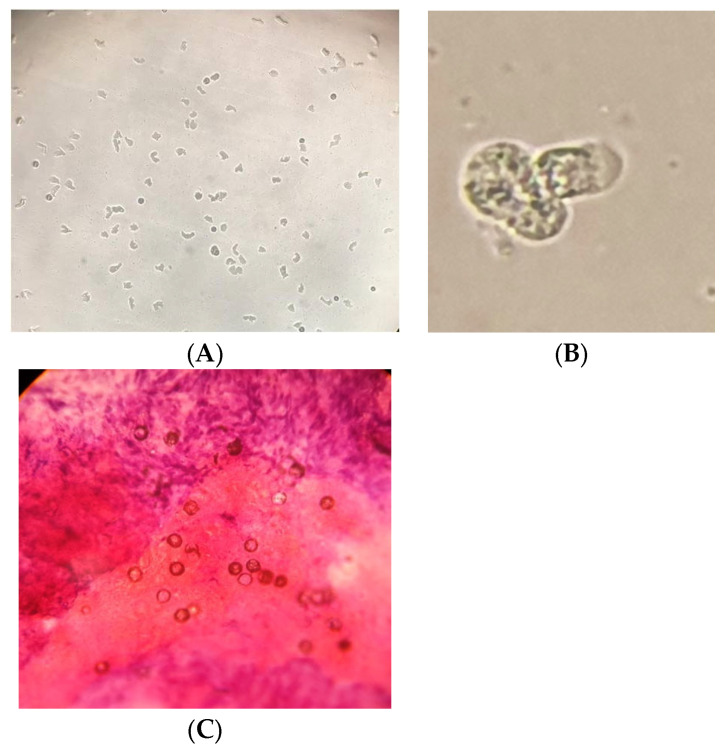
Representative images showing (**A**) *Acanthamoeba* trophozoites and cysts on non-nutrient agar plates (10× magnification),(**B**) *Naegleria fowleri* trophozoites in a wet-mount preparation (40× magnification), and (**C**) Cysts of *Balamuthia mandrillaris* seen in the histopathology of brain tissue (40× magnification).

**Figure 5:**
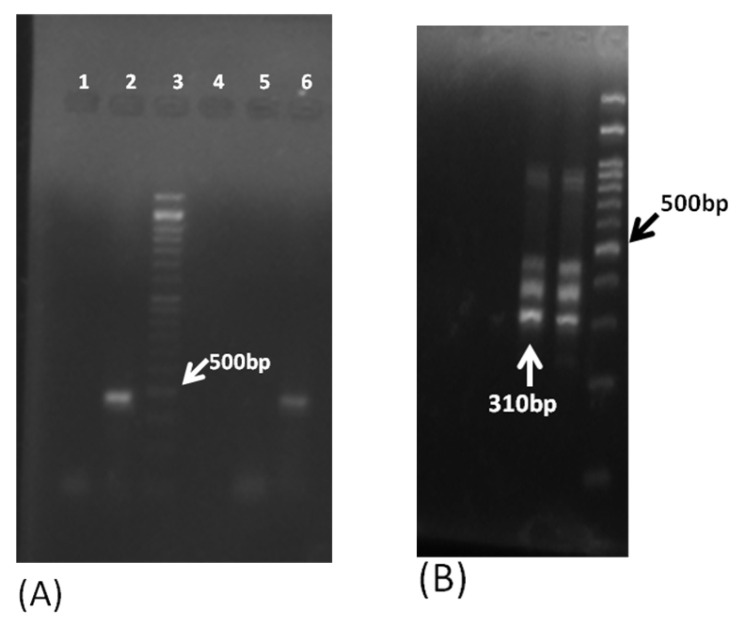
Representative images of 1.5% agarose gel electrophoresis for the PCR targeting the internal transcribed sequence (ITS) region of *Naegleria* spp. and *N. fowleri.* (**A**) Lane 1, negative control; lane 2, positive control for *Naegleria* spp. amplifying band between 408–457 bp (first cycle); lane 3, 100 bp molecular marker; lanes 4 and 5, samples found negative for *Naegleria* spp.; lane 6, sample found positive for *Naegleria* spp. (**B**) Lane 1, negative control; lane 2, samples found negative for *N. fowleri*; lane 3, positive sample for *N. fowleri*; lane 4, positive control for *N. fowleri* amplifying a band of 310 bp (second cycle); lane 5, 100 bp molecular marker.

In addition to the PCR-based detection, all 11 samples were found to be positive by the respective confirmatory tests, i.e., culture on NNA/microscopy/histopathology in the case of *Acanthamoeba* spp. and *N. fowleri* and histopathology of the tissue biopsies for *B. mandrillaris* (Figure 4). The six *Acanthamoeba*-positive samples were successfully isolated on the NNA plates. Of the two *N. fowleri* samples, only one was successfully isolated on the NNA medium, possibly due to the limited quantity of the CSF sample. However, *N. fowleri* was identified microscopically in this sample and confirmed by PCR. The three *B. mandrillaris*-positive samples (which cannot be isolated in a culture medium) were confirmed by histopathological examination. The eight pan-FLA PCR-positive samples were further confirmed by the parasitic-specific PCR, which confirmed six samples positive for *Acanthamoeba* spp. (Figure 2A) and two for *N. fowleri* (Figure 5).

The six *Acanthamoeba* spp.-positive samples included five CSF samples and one brain abscess sample. The *N. fowleri* was found positive in CSF samples, and *B. mandrillaris* was detected in brain tissue biopsies. The diagnostic characteristics of parasite-specific PCRs were evaluated using culture on NNA/microscopy/histopathology as the confirmatory test for *Acanthamoeba* spp. and *N. fowleri* and histopathology for *B. mandrillaris.* The sensitivity, specificity, PPV, and NPV of the different diagnostic methods for *Acanthamoeba* spp., *N. fowleri*, and *B. mandrillaris* was 100%, and the inter-rater reliability of culture/microscopy/histopathology and PCR for *Acanthamoeba* spp. and *N. fowleri* and histopathology and PCR for *B. mandrillaris* was one, indicating 100% agreement between all tests evaluated in this study (Table 2).

The *B. mandrillaris*-specific mitochondrial *16S rDNA* and *18S rDNA* primers were used in a PCR that included known positive samples for *T. gondii*, *E. histolytica*, and *N. fowleri.* The use of mitochondrial *16S rDNA* gene primers showed multiple bands of variable sizes in samples previously found positive for *T. gondii*, *E. histolytica*, and *N. fowleri*, representing a certain amount of cross reactivity, leading to confusion, although no band at the position of *B. mandrillaris* was observed (Lane 2–4 in Figure 3A). In contrast, the primers targeting the *18S rDNA* gene of *B. mandrillaris* did not produce any non-specific bands (Figure 3B). Lanes 14 and lane 16 in Figure 3B represent the amplicons of the positive control from the second and first PCR, respectively. The other disease controls produced no amplification. The *B. mandrillaris*-positive control produced an amplification product of 403 bp and 201 bp from the first and second PCR cycle, respectively, as expected (Figure 3B).

## 4. Discussion

Free-living amoebae, viz., *Acanthamoeba*, *N. fowleri*, and *B. mandrillaris* can cause a wide range of infections, such as keratitis [24] and CNS infections [25,26]. CNS infections due to FLA are mainly caused by various species of *Acanthamoeba*, *B. mandrillaris,* and *N. fowleri* and often go neglected, mainly due to the lack of awareness and delays in disease diagnosis. The poor sensitivity of microscopic examination warrants specific and sensitive molecular assays for detection of CNS infections. PCR assays can detect pathogens efficiently in clinical samples with good sensitivity and specificity and are affordable.

In the present study, we evaluated PCRs targeting different genetic regions in patient samples suspected of encephalitis/meningoencephalitis for the detection of FLA. Initial screening was conducted by pan-FLA PCR, followed by parasitic-specific PCR for the detection of FLA in CSF, brain biopsy, and brain abscess samples. All the samples were evaluated by confirmatory tests: culture on NNA for *Acanthamoeba* spp. and *N. fowleri*. The histopathological examination was considered the gold standard for *B. mandrillaris*-positive samples, as it does not grow on NNA plates. Although axenic cultivation of *B. mandrillaris* has been reported, it is tedious and time-consuming to cultivate each patient sample axenically [27]. 

We evaluated 156 patient CNS samples and found 11 positive samples, including 6 *Acanthamoeba* spp., 3 *B. mandrillaris,* and 2 *N. fowleri*. In comparison to the respective confirmatory methods, the sensitivity, specificity, PPV, and NPV were 100% for PCR-based detection of *Acanthamoeba* spp., *N. fowleri*, and *B. mandrillaris*. The inter-rater reliability between the PCRs and the confirmatory tests for the detection of *Acanthamoeba* spp., *N. fowleri*, and *B. mandrillaris* was one, indicating 100% agreement between the diagnostic assays. The results from the present study support the deployment of PCR-based assays for the detection of FLA in clinical samples. We previously reported cases of *B. mandrillaris* and *Acanthamoeba* spp. and one case of *N. fowleri* [28,29,30,31,32], which were confirmed by molecular assays. In addition, we previously reported the diagnostic performance of *Acanthamoeba* JDP primers targeting the *18S rRNA* gene for the diagnosis of *Acanthamoeba* keratitis [33]. The primers targeting the *Acanthamoeba* spp.-specific 18S rRNA gene and the 18S rDNA and mitochondrial 16S rRNA gene of *B. mandrillaris* have been previously evaluated for their sensitivity [21,23,33,34].

As the pan-FLA PCR primers used routinely for detection of *Acanthamoeba* spp. and *N. fowleri* do not include *B. mandrillaris* as a target, it is important to use an assay that is specific and sensitive in detecting *B. mandrillaris*. Previously, primers based on mitochondrial the *16S rRNA* gene have been widely used for this purpose [22,34]; however, we observed multiple bands of varying size close to the *B. mandrillaris* target of 1075 bp using this PCR (Figure 3A). These multiple bands tend to create confusion in the visual interpretation of results. Yagi et al. [34] reasoned that DNA from the leukocytes or necrotic tissue might be the reason for such multiple bands. We observed smearing on the gel, especially in tissue biopsy samples when using primers targeting the mitochondrial *16S rRNA* gene of *B. mandrillaris.* Therefore, in the present study, we evaluated the *18S rDNA* gene-nested primers for *B. mandrillaris* reported by Ahmad et al. [23] on our samples. In addition to being 100% sensitive, multiple bands were not observed in the CSF, brain biopsy, or brain abscess samples, and the samples positive for *T. gondii*, *E. histolytica*, and *N. fowleri* were found negative for *B. mandrillaris*. A probable reason for the improved specificity could be the nested PCR with a higher annealing temperature for the first PCR cycle, eliminating non-specific annealing. Thus, this target may be preferred for detection of *B. mandrillaris* in clinical samples.

In conclusion, we found the use of PCRs an efficient strategy for the detection of FLA in patients suspected of encephalitis/meningoencephalitis in this study. Generally, separate PCRs are performed for detection *Acanthamoeba* spp., *N. fowleri*, and *B. mandrillaris*; however, this is time-consuming and prohibitory for many labs. Based on our experience, we suggest using pan-FLA PCR to first identify *Acanthamoeba* spp. and *N. fowleri* in a single reaction and separately performing *18S rDNA* gene-nested PCR for *B. mandrillaris*, thus conserving time and resources. CNS afflictions caused by FLAs remain a diagnostic challenge, owing to a lack of awareness and diagnostic capabilities of laboratories. Because FLA infections of the CNS are only sporadically diagnosed, it is difficult to carry out large-scale evaluations of molecular assays for these parasites. An alternative approach to accumulate data on the robustness of these tests is the use of these PCRs by molecular laboratories across the world so that such data can be accumulated and utilized to design evidence-based diagnostic strategies for the timely diagnosis and management of fatal FLA infections of the CNS.

## Figures and Tables

**Table 1:** Details of the oligonucleotides used in the present study.

	Forward (5′–3′)	Reverse (5′–3′)	Product Size (bp)		Reference
Housekeeping GAPDH gene *					[18]
	GAAGGTGAAGGTCGGAGTCAAC	CAGAGTTAAAAGCAGCCCTGGT	380		
Pan-FLA 18S rRNA gene					[19]
	CGCGGTAATTCCAGCTCCAATAGC	CAGGTTAAGGTCT CGTTCGTTAAC	800	*Hartmanella vermiformis*	
			900	*Naegleria fowleri*	
			950	*Vannella* sp., *Vahlkampfia ovis*	
			1080	*A.castellanii*, *A. polyphaga*, *A. lenticulata*, *A. hatchetti*	
			1350	*A. comadoni*	
			1500	*A. astronyxis*	
*Acanthamoeba* spp.-specific 18S rRNA gene (JDP gene)					[20]
	GGCCCAGATCGTTTACCGTGAA	TCTCACAAGCTGCTAGGGAGT	450–500		
*Naegleria* spp.- and *Naegleria fowleri*-specific primers targeting internal transcribed sequences (ITS1 and ITS2)					[21]
First cycle (Genus specific)	GAACCTGCGTAGGGATCATTT	TTTCTTTTCCTCCCCTTATTA	408–457		
Second cycle	GTGAAAACCTTTTTTCCATTTACA	AAATAAAAGATTGACCATTTGAAA	310		
(Species-specific)					
Mitochondrial 16S rRNA gene of *Balamuthia mandrillaris*					[22]
	CGCATGTATGAAGAAGACCA	TTACCTATATAATTGTCGATACCA	1075		
18S rDNA gene of *Balamuthia mandrillaris*					[23]
First cycle	GGTTCGTGCCCCTTGCCTTCTG-3′	GGTTCGTGCCCCTTGCCTTCTG	403		
Second cycle	GGTTCGTGCCCCTTGCCTTCTG-3′	GGTCGAGCTCCGAA	201		

* The GAPDH gene was used as a housekeeping gene in all the patient samples to evaluate the successful DNA extraction and amplification of genomic DNA.

**Table 2:** The diagnostic characteristics and the inter-rater reliability score of the different diagnostic techniques for the detection of *Acanthamoeba* spp., *Naegleria fowleri*, and *Balamuthia mandrillaris*.

Parameter	Total Number of Positive Samples	Sensitivity (95% CI)	Specificity (95% CI)	PPV	NPV	Cohen’s Kappa Coefficient Test (Inter-Rater Reliability Score)
*Acanthamoeba* spp.						
Microscopy/histopathology	6	100% (47.82–100)	100% (97.57–100)	100%	100%	*k* = 1 100% agreement
Culture on NNA	6	100% (47.82–100)	100% (97.57–100)	100%	100%	
Pan-FLA PCR	6	100% (47.82–100)	100% (97.57–100)	100%	100%	
*18S rRNA* gene-specific JDP PCR	6	100% (47.82–100)	100% (97.57–100)	100%	100%	
*Naegleria fowleri*						
Microscopy/ histopathology	2	100% (15.81–100)	100% (15.81–100)	100%	100%	*k* = 1 100% agreement
Culture on NNA	1 *	100% (15.81–100)	100% (15.81–100)	100%	100%	
Pan-FLA PCR	2	100% (15.81–100)	100% (97.62–100)	100%	100%	
(ITS 1 and ITS 2) gene-specific PCR	2	100% (15.81–100)	100% (97.62–100)	100%	100%	
*Balamuthia mandrillaris*						
Histopathological examination	3	100% (29.24–100)	100% (97.60–100)	100%	100%	*k* = 1 100% agreement
Mitochondrial *16S rRNA* gene-specific PCR	3	100% (29.24–100)	100% (97.60–100)	100%	100%	
*18S rRNA* gene-specific PCR	3	100% (29.24–100)	100% (97.60–100)	100%	100%	

CI: confidence interval; PCR: polymerase chain reaction; NNA: non-nutrient agar; NPV: negative predictive value; PPV: positive predictive value. * Due to the limited quantity of CSF samples, NNA culture was not performed. CSF was used for microscopy and PCR-based detection.

## Data Availability

All the relevant data has been incorporated in the article.
